# Supplementary material for: Heat adaptation of phage T7 under an extended genetic code
Source: Virus Evol. 2021 Dec 1;7(2):veab100. doi: 10.1093/ve/veab100 (PMC8923235; doi:10.1093/ve/veab100)
Supplement: veab100_Supp [file veab100_supp.zip › Sequencing_Analysis.zip!/Sequencing_Analysis/A3/evidence/JC_SIDE_2_61.html]

BRESEQ :: Evidence


| New junction evidence | | | | | | | | | | | |
| --- | --- | --- | --- | --- | --- | --- | --- | --- | --- | --- | --- |
|  | | seq id | position | reads (cov) | reads (cov) | score | skew | freq | annotation | gene | product |
| \* | ? | NC\_001604.1 | = 7613 | 2216 (1.270) | 19 (0.010) | 11/586 | 38.5 | 0.93% | intergenic (–/–) | *–/–* | –/– |
|  | ? | NC\_001604.1 | 7912 = | 1900 (1.110) |  |  |  |  | intergenic (–/–) | *–/–* | –/– |
| Rejected: Coverage evenness skew score above cutoff. | | | | | | | | | | | |
| Rejected: Frequency below/above cutoff threshold. | | | | | | | | | | | |

Only 100 of 1918 total aligned reads displayed.  

`TTAAGAAGGTTGGTAAATTCCTTGCGGCTTTGGCAGCTATCCTGACGCTTGCGTATATTCTTGCGGTATACCCTCAAGTAGCACTAGTAGTAGTTGGCGCTTGTTACTTAGCGGCAGTGTGTGCTTGCGTGTGGAGTATAGTTAACTGGTAATACGACTCACTAAAGGAGGTACACACCATGATGTACTTAATGCCATTACTCATCGTCATTGTAGGATGCCTTGCGCTCCACTGTAGCGATGATGATATGCCAGATGGTCACGCTTAATACGACTCACTAAAGGAGACA‑CTATATGTTTCGACTTCATTACAACAAAAGCGTTAAGAATTTCACGGTTCGCCGTGCTGACCGTTCAATCGTATGTGCGAGCGAGCGCCGAGCTAAGATACCTCTTATTGGTAACACAGTTCCTTTGGCACCGAGCGTCCACATCATTATCACCCGTGGTGACTTTGAGAAAGCAATAGACAAGAAACGTCCGGTTCTTAGTGTGGCAGTGACCCGCTTCCCGTTCGTCCGTCTGTTACTCAAACGAATCAAGGAGGTGTTCTGATGGGACTGTTAGATGGTGAAGCCTGGGAAAAAGAAAACCCGCC  >  NC_001604.1/7612‑8209`  

                                                                                                                                                                                                                                                                                                             `|`                                                                                                                                                                                                                                                                                                           
                 `ttCCTTGCGGCTTTGGCAGCTATCCTGACGCTTGCGTATATTCTTGCGGTATACCCTCAAGTAGCACTAGTAGTAGTTGGCGCTTGTTACTTAGCGGCAGTGTGTGCTTGCGTGTGGAGTATAGTTAACTGGTAATACGACTCACTAAAGGAGGTACACACCATGATGTACTTAATGCCATTACTCATCGTCATTGTAGGATGCCTTGCGCTCCACTGTAGCGATGATGATATGCCAGATGGTCACGCTTAATACGACTCACTAAAGGAGACA‑CTATATGTTTCGACTTCATTACAACaaa                                                                                                                                                                                                                                                                                          >  1:251904/1‑301 (MQ=255)`  
                   `ccTTGCGGGTTTGGCAGCTATCCTGACGCTTGCGTATATTCTTGCGGTATACCCTCAAGTAGCACTAGTAGTAGTGGGCGCTTGTTACTTAGCGGCAGTGTGTGCTTGCGTGTGGAGTATAGTTAACTGGTAATACGACTCACTAAAGGAGGTACACACCATGATGTACTTAATGCCATTACTCATCGTCATTGTAGGATGCCTTGCGCTCCACTGTAGCGATGATGATATGCCAGATGGTCACGCTTAATACGACTCACTAAAGGAGACA‑CTATATGTTTCGACTTCATTACAACAAAAg                                                                                                                                                                                                                                                                                        <  1:203530/301‑1 (MQ=255)`  
                           `gtgtGGCAGCTATCCGGACGCTTGTGTATATTCGTGCGGTAGACCATCAAGTAGCACTAGTAGGAGTTGGCGCTTGTTACTTAGCGGAAGTGTGGGCTTGCGTGTGGAGTATAGTTAACTGGTAATACGACTCACTAAAGGAGGTACACACCATGATGTACTTAATGCCATTACTCATCGTCATTGTAGGATGCCTTGCGCTCCACTGTAGCGATGATGAGAGGCCAGATGGTCACGCTTAATACGACTCACTAAAGGAGACA‑CTATATGTTTCGACTTCATTACAACAAAAGCGTTAAGa                                                                                                                                                                                                                                                                                <  1:26939/298‑1 (MQ=255)`  
                           `cTTTGGCTGCTATCCTGACGATTGCGTATATTCTTGAGGTATACCCTCAAGTAGCACTAGTAGTAGTTGGAGCTTATTACTTAGCGGCAGTGTGTGCTTGCGTGTGGAGTATAGTTAACTGGTAATACGACTCACTAAAGGAGGTACAAACCATGATGTACTTAATGCCATTACACATCGTCATTGTAGGATGCCTTGCGCTCCACTGTAGCGATGATGATATGCCAGATGGTCACGCTTAATACGACTCACTAAAGGAGACA‑CTATATGTTTCGACTTCATTACAACAAAAGCGTTAAGa                                                                                                                                                                                                                                                                                <  1:329343/301‑1 (MQ=255)`  
                           `ataagagacagaTCCTGACGCTTGGGTATATTCTTGCGGTATACCCTCAAGTAGCACTAGTAGTAGTTGGCGCTTGTCACTTATCGGCAGTGTGTGCTTGCGTGTGGAGTATAGTTAACTGGTAATACGACTCACTAAAGGAGGTACACACCATGATGTACTTAATGCCATTACTCATCGTCATTGTAGGATGCCTTGCGCTCCACTGTAGCGATGATGATATGCCAGATGGTCACGCTTAATACGACTCACTAAAGGAGACA‑CTATATGTTTCGACTTCATTACAACAAAAGCGTTAAGa                                                                                                                                                                                                                                                                                <  1:219473/290‑1 (MQ=255)`  
                                              `gtgggctcggagatgtgtataagagacagaaGTAGCACTAGTAGTAGTTGGCGCTTGTTACTTAGCGGCAGTGTGTGCTTGCGTGTGGAGTATAGTTAACTGGTAATACGACTCACTAAAGGAGGTACACACCATGATGTACTTAATGCCATTACTCATCGTCATTGTAGGATGCCTTGCGCTCCACTGTAGCGATGATGATATGCCATATGGTCACGCTTAATACGACTCACTAAAGTAGACA‑CTATATGTTTCGACTTCATTACAACAAAAGCGTTAATAATTTCACGGTTCGCCGTGc                                                                                                                                                                                                                                                             <  1:163537/272‑1 (MQ=255)`  
                                               `cTTGCGTATATTCTTGCGGTATACCCTCAAGTAGCACTAGTAGTAGTTGGCGCTTGTTACTTAGCGGCAGTGTGTGCTTGCGTGTGGAGTATAGTTAACTGGTAATACGACTCACTAAAGGAGGTACACACCATGATGTACTTAATGCCATTACTCATCGTCATTGTAGGATGCCTTGCGCTCCACTGTAGCGATGATGATATGCCAGATGGTCACGCTTAATACGACTCACTAAAGGAGACA‑CTATATGTTTCGACTTCATTACAACAAAAGCGTTAAGAATTTCACGGTTCGACGTGCt                                                                                                                                                                                                                                                            >  1:261825/1‑301 (MQ=255)`  
                                                  `gCGTATATTCTTGTGGTATACCCTCAAGTAGCACTAGTAGTAGTTGGCGCTTGTTACTTAGCGGCAGTGTGTGCTTGCGTGTGGAGTATAGTTAACTGGTAATACGACTCACTAAAGGAGGTACACACCATGATGTACTTAATGCCATTACTCATCGTCATTGTAGGATGCCTTGCGCTCCACTGTAGCGATGATGATATGCCAGATGGTCACGCTTAATACGACTCACTAAAGGAGACA‑CTATATGTTTCGACTTCATTACAACAAAAGCGTTAAGAATTTCACGGTTCGCCGTGCTGAc                                                                                                                                                                                                                                                         >  1:68994/1‑301 (MQ=255)`  
                                                    `gTATATTCTTGCGGTATACCCTCAAGTAGCACTAGTAGTAGTTGGCGCTTGTTACTTAGCGGCAGTGTGTGCTTGCGTATGGAGTATAGTTAACTGGTAATACGACTCACTAAAGGAGGTACACACCATGATGTACTTAATGCCATTACTCATCGTCATTGTAGGATGCCTTGCGCTCCACTGTAGCGATGATGATATGCCAGATGGTCACGCTTAATACGACTCACTAAAGGAGACA‑CTATATGTTTCGACTTCATTACAACAAAAGCGTTAAGAATTTCACGGTTCGCAGTGCTGACCg                                                                                                                                                                                                                                                       >  1:258677/1‑301 (MQ=255)`  
                                                       `tatTCTTGCGGTATACCCTCAAGTAGCACTAGTAGTAGTTGGCGCTTGTTACTTAGCGGCAGTGTGTGCTTGCGTGTGGAGTATAGTTAACTGGTAATACGACTCACTAAAGGAGGTACACACCATGATGTACTTAATGCCATTACTCATCGTCATTGTAGGATGCCTTGCGCTCCACTGTAGCGATGATGATATGCCAGATGGTCACGCTTAATACGACTCACTAAAGGAGACA‑CTATATGTTTCGACTTCATTACAACAAAAGCGTTAAGAATTTCACGGTTCGCCGTGCTGACCGTTc                                                                                                                                                                                                                                                    >  1:243593/1‑301 (MQ=255)`  
                                                                            `aGTAGCACTAGTAGTAGTTGGCGCTTGTTACTTAGCGGCAGTGTGTGCTTGCGTGTGGAGTATAGTTAACTGGTAATACGACTCACTAAAGGAGGTACACACCATGATGTACTTAATGCCATTACTCATCGTCATTGTAGGATGCCTTGCGCTCCACTGTAGCGATGATGATATGCCAGATGGTCACGCTTAATACGACTCACTAAAGGAGACA‑CTATATGTTTCGACTTCATTACAACAAAAGCGTTAAGAATTTCACGGTTCGCCGTGCTGACTGTTCAATCGTATGTGCGagtgagcg                                                                                                                                                                                                                               >  1:91503/1‑301 (MQ=255)`  
                                                                                `gCACTAGTAGTAGTTGGCGCTTGTTACTTAGCGGCAGTGTGTGCTTGCGTGTGGAGTATAGTTAACTGGTAATACGACTCACTAAAGGAGGTACACACCATGATGTACTTAATGCCATTACTCATCGTCATTGTAGGATGCCTTGCGCTCCACTGTAGCGATGATGATATGCCAGATGGTCACGCTTAATACGACTCACTAAAGGAGACA‑CTATATGTTTCGACTTCATTACAACAAAAGCGTTAAGAATTTCACGGTTCGCCGTGCTGACCGTTCAATCGTATGTGCGAGCGAGCGCCGa                                                                                                                                                                                                                           >  1:222061/1‑301 (MQ=255)`  
                                                                                    `tagtagtagTTGGCGCTTGTTACTTAGCGGCAGTGTGTGCTTGCGTGTGGAGTATAGTTAACTGGTAATACGACTCACTAAAGGAGGTACACACCATGATGTACTTAATGCCATTACTCATCGTCATTGTAGGATGCCTTGCGCTCCACTGTAGCGATGATGATATGCCAGATGGTCACGCTTAATACGACTCACTAAAGGAGACA‑CTATATGTTTCGACTTCATTACAACAAAAGCGTTAAGAATTTCACGGTTCGCCGTGCTGACCGTTCAATCGTATGTGCGAGCGAGCGCCGAGCTa                                                                                                                                                                                                                       >  1:140291/1‑301 (MQ=255)`  
                                                                                    `tagtagtagTTGGCGCTTGTTACTTAGCGGCAGTGTGTGCTTGCGTGTGGAGTATAGTTAACTGGTAATACGACTCACTAAAGGAGGTACACACCATGATGTACTTAATGCCATTACTCATCGTCATTGTAGGATGCCTTGCGCTCCACTGTAGCGATGATGATATGCCAGATGGTCACGCTTAATACGACTCACTAAAGGAGACA‑CTATATGTTTCGACTTCATTACAACAAAAGCGTTAAGAATTTCACGGTTCGCCGTGCTGACCGTTCAATAGTAtgctgtatcttatacacatctc                                                                                                                                                                                                                       >  1:94927/1‑281 (MQ=255)`  
                                                                                     `agtagtagtTGGCGCTTGTTACTTAGCGGCAGTGTGTGCTTGCGTGTGGAGTATAGTTAACTGGTAATACGACTCACTAAAGGAGGTACACACCATGATGTACTTAATGCCATTACTCATTGTCATTGTAGGATGCCTTGCGCTCCACTGTAGCGATGATGATATGCCAGATGGTCACGCTTAATACGACTCACTAAAGGAGACA‑CTATATGTTTCGACTTCATTACAACAAAAGCGTTAAGAATTTCACGGTTCGCCGTGCTGACCGTTCAATCGTATGTGCGAGCGAGCGCCGAGCTaa                                                                                                                                                                                                                      >  1:181986/1‑301 (MQ=255)`  
                                                                                     `agtagtagtTGGCGCTTGTTACTTAGCGGCAGTGTGTGCTTGCGTGTGGAGTATAGTTAACTGGTAATACGACTCACTAAAGGAGGTACACACCATGATGTACTTAATGCCATTACTCATCGTCATTGTAGGATGCCTTGCGCTCCACTGTAGCGATGATGATATGCCAGATTGTCACGCTTAATACGACTCACTAAAGGAGACA‑CTATATGTTTCGACTTCATTACAACAAAAGCGTTAAGAATTTCACGGTTCGCCGTGCTGACCGTTCAATCGTATGTGCGAGCGAGCGCCGAGCtct                                                                                                                                                                                                                      >  1:157575/1‑299 (MQ=255)`  
                                                                                      `gtagtagtTGGCGCTTGTTACTTAGCGGCAGTGTGTGCTTGCGTGTGGAGTATAGTTAACTGGTAATACGACTCACTAAAGGAGGTACACACCATGATGTACTTAATGCCATTACTCATCGTCATTGTAGGATGCCTTGCGCTCCACTGTAGCGATGATGATATGCCAGATGGTCACGCTTAATACGACTCACTAAAGGAGACA‑CTATATGTTTCGACTTCATTACAACAAAAGCGTTAAGAATTTCACGGTACGCCGTGCTGACCGTTCAATCGTATGTGCGAGCGAGCGCCGAGCTAAg                                                                                                                                                                                                                     >  1:212910/1‑301 (MQ=255)`  
                                                                                        `agtagtTGGCGCTTGTTACTTAGCGGCAGTGTGTGCTTGCGTGTGGAGGATAGTTAACTGGTAATACGACTCACTAAATGAGGTACACACCATGATGTACTTAATGCCATTACTCATCGTCATTGTAGGATGCCTTGCGCTCCACTGTAGCGATGATGATATGCCAGATGGTCACGCTTAATACGACTCACTAAAGGAGACA‑CTATATGTTTCGACTTCATTACAACAAAAGCGTTAAGAATTTCACGGTTCGCCGTGCTGACCGTTCAATCGTATGTGCGAGCGAGCGCCGAGCTAAGAt                                                                                                                                                                                                                   <  1:322732/301‑1 (MQ=255)`  
                                                                                        `agtagtTGGCGCTTGTTACTTAGCGGCAGTGTGTGCTTGCATGTGGAGTATAGTTAACTGGTAATACGACTCACTAAAGGAGGTACACACCATGATGTACTTAATGCCATTACTCATCGTCATTGTAGGATGCCTTGCGCTCCACTGTAGCGATGATGATATGCCAGATGGTCACGCTTAATACGACTCACTAAAGGAGACA‑CTATATGTTTCGACTTCATTACAACAAAAGCGTTAAGAATTTCACGGTTCGCCGTGCTGACCGTTCAATCGTATGTGCGAGCGAGCGCCGAGCTAAGAt                                                                                                                                                                                                                   <  1:123502/301‑1 (MQ=255)`  
                                                                                          `gctcggagatgggtataagagacaaggtgtgtGCTTGCGTGTGGAGTATAGTTAACTGGTAATACGACTCACTAAAGGAGGTACACACCATGATGTACGTAATGCCAGTACTCATCGTCATTGTAGGATGCCTTGCGCTCCACTGTAGCGATGATGATATGCCAGATGGTCACGCTTAATACGACTCACTAAAGGAGACA‑CTATATGTTTCGACTTCATTACAACAAAAGCGTTAAGAATTTCACGGTTCGCCGTGCTGACCGTTCAATCGTATGTGCGAGCGAGCGCCGAGCTAAGATAc                                                                                                                                                                                                                 <  1:337172/275‑1 (MQ=255)`  
                                                                                                 `cgcTTGTTACTTAGCGGCAGTGTGTGCGTGCGTGTGGAGTATAGTTAACTGGTAATACGACTCACTAAAGGAGGTACACACCATGATGTACTTAATGCCATTACTCATCGTCATTGTAGGATGCCTTGCGCTCCACTGTAGCGATGATGATATGCCAGATGGTCACGCTTAATACGACTCACTAAAGGAGACA‑CTATATGTTTCGACTTCATTACATCAAAAGCGTTAAGAATTTCACGGTTCGCCGTGCTGACCGTTCAATCGTATGTGCGAGCGAGCGCCGAGCTAAGATACCTCTTAt                                                                                                                                                                                                          <  1:174910/301‑1 (MQ=255)`  
                                                                                                     `tGTTACTTGGCGGCAGTGTGTGCTTGCGTGTGGAGTATAGTTAACTGGTAATACGACTCACTAAAGGAGGTACACACCATGATGTACTTAATGCCATTACTCATCGTCATTGTAGGATGCCTTGCGCTCCACTGTAGCGATGATGATATGCCAGATGGTCACGCTTAATACGACTCACTAAAGGAGACA‑CTATATGTTTCGACTTCATTACAACAAAAGCGTTAAGAATTTCACGGTTCGCCGTGCTGACCGTTCAATCGTATGTGCGAGCGAGCGCCGAGCTAAGATACCTCTTATTGGt                                                                                                                                                                                                      <  1:87966/301‑1 (MQ=255)`  
                                                                                                      `gTTCCTTAGCGGCAGTGTGTGCTTGCGTGTGGAGTATAGTTAACTGGTAATACGACTCACTAAAGGAGGTACACACCATGATGTACTTAATGCCATTACTCATCGTCATTGTAGGATGCCTTGCGCTCCACTGTAGCGATGATGATATGCCAGATGGTCACGCTTAATACGACTCACTAAAGGAGACA‑CTATATGTTTCGACTTCATTACAACCAAAGCGTTAAGAATTTCACGGTTAGCCGTGCTGACCGTTCAATCGTATGTGCGAGCGTGCGCCGAGCTAAGATACCTcttcttctgt                                                                                                                                                                                                     >  1:169113/1‑297 (MQ=255)`  
                                                                                                      `gTTACTTAGCGGCAGTGTGTGCTTGCGTGTGGAGTATAGTTAACTGGTAATACGACTCACTAAAGGAGGTACACACCATGATGTACTTAATGCCATTACTCATCGTCATTGTAGGATGCCTTGCGCTCCACTGTAGCGATGATGATATGCCAGATGGTCACGCTTAATACGACTCACTAAAGGAGACA‑CTATATGTTTCGACTTCATTACAACAAAAGCGTTAAGAATTTCACGGTTCGCCGTGCTGACCGTTCAATCGTATGTGCGAGCGAGCGCCGAGCTAAGATACCTCTTATTGGTa                                                                                                                                                                                                     >  1:255800/1‑301 (MQ=255)`  
                                                                                                          `gttAGCGGCAGTGTGTGCTTGCGTGTGGAGTATAGTTAACTGGTAATACGACTCACTAAAGGAGGTACACACCATGATGTACTTAATGCCATTACTCATCGTCATTGTAGGATGCCTTGCGCTCCACTGTAGCGATGATGATATGCCAGATGGTCACGCTTAATACGACTCACTAAAGGAGACA‑CTATATGTTTCGACTTCATTACAACAAAAGCGTTAAGAATTTCACGGTTCGCAGTGCTGACCGTTCAATCGTATGTGCGAGCGAGCGCCGAGCTAAGATACCTCTTATTGGTAacac                                                                                                                                                                                                 <  1:59634/300‑1 (MQ=255)`  
                                                                                                               `gacaggtgtgtGCTTGCGTGTGGAGTATAGTTAACTGGTAATACGACTCACTAAAGGAGGTACACACCATGATGTACTTAATGCCATTACTCATCGTCATTGTAGGATGCCTTGCGCTCCACTGTAGCGATGATGATATGCCAGATGGTCACGCTTAATACGACTCACTAAAGGAGACA‑CTATATGTTTCGACTTCATTACAACAAAAGCGTTAAGAATTTCACGGTTCGCCGTGCTGACCGTTCAATCGTATGTGCGAGCGAGCGCCGAGCTAAGATACCTCTTATTGGAAACACAGTTc                                                                                                                                                                                            <  1:133930/296‑1 (MQ=255)`  
                                                                                                                             `tGCGTGTGGAGTATAGTTAACTGGTAATACGACTCACTAAAGGAGGTACACACCATGATGTACTTAATGCCATTACTCATCGTCATTGTAGGATGCCTTGCGCTCCACTGTAGCGATGATGATATGCCAGATGGTCACGCTTAATACGACTCACTAAAGGAGACA‑CAATATGTTTCGACTTCATTACAACAAAAGCGTTAAGAATTTCACGGTTCGCCGTGCGGACCGTTCAATCGTATGTGCGAGCGAGCGCCGAGCTAAGATACCTCTTATTGGTaaacaagctgacccttatacaaaa                                                                                                                                                                              >  1:100162/1‑279 (MQ=255)`  
                                                                                                                               `cGTGTGGAGTATAGTTAACTGGTAATACGACTCACTAAAGGAGGTACACACCATGATGTACTTAATGCCATTACTCATCGTCATTGTAGGATGCCTTGCGCTCCACTGTAGCGATGATGATATGCCAGATGGTCACGCTTAATACGACTCACTAAAGGAGACA‑CTATATGTTTCGACTTCATTACAACAAAAGCGTTAAGAATTTCACGGTTCGCCGTGCTTACCGTTCAATCGTATGTTCGAGCGAGCGCCGAGCTAAGATACCTCTTCTTGGTAACACAGTTCCTTTGGCACCGAGCGt                                                                                                                                                                            >  1:129887/1‑301 (MQ=255)`  
                                                                                                                                    `caggTATAGTTAACTGGTAATACGACTCACTAAAGGAGGTACACACCATGATGTACTTAATGCCATTACTCATCGTCATTGTAGGATGCCTTGCGCTCCACTGTAGCGATGATGATATGCCAGATGGTCACGCTTAATACGACTCACTAAAGGAGACA‑CTATACGTTTCGACTTCATTACAACAAAAGCGTTAAGAATTTCACAGTTCGCCGTGCTGACAGTTCAATCGTATGTGCGAGCGAGCGCCGAGCTAAGATACCTCTTATTGGTAACACAGTTCCTTTGGCACCGAGCGTCcaca                                                                                                                                                                       <  1:137260/298‑1 (MQ=255)`  
                                                                                                                                     `gAGTATAGTTAACTGGTAATACGACTCACTAAAGGAGGTACACACCATGATGTACTTAATGCCATTACTCATCGTCATTGTAGGATGCCTTGCGCTACACTGTAGCGATGATGATATGCCAGATGGTCACGCTTAATACGACTAACTAAAGGAGACA‑CTATATGTTTCGACTTCATTACAACAAAAGCGTTAAGAATTTAACGGTTCGCCGTGCTGACCGTTCAATCGTATGTGCGAGCGAGCGCCGAGCTAAGATACCTCTTATTGGTAACACAGTTCCTTTGGCACCGAGCGTCCAcat                                                                                                                                                                      >  1:316020/1‑301 (MQ=255)`  
                                                                                                                                      `acagtaGTTAACTGGTAATACGACTCACTAAAGGAGGTACACACCATGATGTACTTAATGCCATTACTCATCGTCATTGTAGGATGCCTTGCGCTCCACTGTAGCGATGATGATATGCCAGATGGTCACGCTTAATACGACTCACTAAAGGAGACA‑CTATATGTTTCGACTTCATTACAACAAAAGCGTTAAGAATTTCACGGTTCGCCGTGCTGACCGTTCAATCGTATGTGCGAGCGAGCGCCGAGCTAAGATACCTCTTATTGGTAACACAGTTCCTTTGGCACCGAGCGTCCAcatc                                                                                                                                                                     <  1:288694/297‑1 (MQ=255)`  
                                                                                                                                       `gTATAGTTAACTGGTAATACGACTCACTAAAGGAGGTACACACCATGATGTACTTAATGCCATTACTCATCGTCATTGTAGGATGCCTTGCGCTCCACTGTAGCGATGATGATATGCCAGATGGTCACGCTTAATACGACTCACTAAAGGAGACA‑CTATATGTTTCGACTTCATTACAACAAAAGCGTGAAGAATTTCACGTTTCGCCGTGCTGACCGTTCAATCGTATGTGCGAGAGAGCGCCGAACTAAGATACCTCTTATTGGTAACAAAGTTCCTTTGGCACCGAGCGTCCAcatac                                                                                                                                                                    >  1:340547/1‑299 (MQ=255)`  
                                                                                                                                           `aGTTAACTGGTAATACGACTCACTAAATGAGGTACACACCATGATGTACTTAATGCCATTACTCATCGTCATTGTAGGATGCCTTGCGCTCCACTGTAGCGATGATGATATGCCAGATGGTCACGCTTAATACGACTCACTAAAGGAGACA‑CTATATGTTTCGACTTCATTACAACAAAAGCGTTAAGAATTTCACGGTTCGCCGTGCTGACCGTTCAATCGTATGTGCGAGCGAGCGCCGAGCTAAGATACCTCTTATTGGTAACACAGTTCCTTTGGCACCGAGCGTCCACATCATTAt                                                                                                                                                                <  1:111045/301‑1 (MQ=255)`  
                                                                                                                                               `aaCTGGTAATACGACTCACTAAAGGAGGTACACACCATGATGTACTTAATGCCATTACTCATCGTCATTGTAGGATGCCTTGCTCTCCACTGTAGCGATGATGATATGCCAGATGGTCACGCTTAATACGACTCACTAAAGGAGACA‑CTATATGTTTCGACTTCATTACAACAAAAGCGTTAAGAATTTCACGGTTCGCCGTGCTGACCGTTCAATCGTATGTGCGAGCGAGCGCCGAGCTAAGATACCTCTTATTGGTAACACAGTTCCTTTGGCACCGAGCGTCCACATCATTATCAcc                                                                                                                                                            <  1:66408/301‑1 (MQ=255)`  
                                                                                                                                                `gtgtataagagaaagcaccagaggcggTACACACCATGATGTACTTAAAGCCATTACTCATCGTCATTGTAGGATGCCTTGCGCTCCACTGTAGCGATGATGATATGCCAGATGGTCACGCTTAATACGACTCACTAAAGGAGACA‑CTATATGTTTCGACTTCATTACAACAAAAGCGTTAAGAATTTCACGGTTCGCCGTGCTGACCGTTCAATCGTATGTGCGAGCGAGCGCCGAGCTAAGATACCTCTTATTGGTAACACAGTTCCTTTGGCACCGAGCGTCCACATCATTATCAccc                                                                                                                                                           <  1:310001/280‑1 (MQ=255)`  
                                                                                                                                                    `gTAATACGACTCACTAAAGGAGGTACACACCATGATGTACTTAATGCCGTTACTCATCGTCATTGTAGGATGCCTTGCGCTCCACTGTAGCGATGATGATATGCCAGATGGTCACGCTTAATACGACTCACTAAAGGAGACA‑CTATATGTTTCGACTTCATGACAACAAAAGCGTTAAGAATTTCACGGTTCGCCGTGCTGACCGTTCAATCGTATGTTCGAGCGAGCGCCGAGCTAAGATACCTCTTATTGGTAACACAGTTCCTTTGGCACCGAGCGTCCACATCATTATCACCCgtgg                                                                                                                                                       <  1:53856/301‑1 (MQ=255)`  
                                                                                                                                                    `gTAATACGACTCACTAAAGGAGGTACACACCATGATGTACTTAATGCCATTACTCATCGTCATTGTAGGATGCCTTGCGCTCCACTGTAGCGATGATGATATGCCAGATGGTCACGCTTAATACGACTCACTAAAGGAGACA‑CTATATGTTTCGACTTCATTACAACAAAAGCGTTAAGAATTTCACGGTTCGCCGTGCTGACCGTTCAATCGTCTGTGCGAGCGAGCGCCGAGCTAAGATACCTCTTATTGGTAACACAGTTCCTTTGGCACCGAGCGTCCACATCATTATCACCCgtgg                                                                                                                                                       >  1:48672/1‑301 (MQ=255)`  
                                                                                                                                                     `tAATACGACTCACTAAAGGAGGTACACACCATGATGTACTTAATGCCATTACTCATCGTCATTGTAGGATGCCTTGCGCTCCACTGTAGCGATGATGATATGCCAGATGGTCACGCTTAATACGACTCACTAAAGGAGACA‑CTATATGTTTCGACTTCATTACAACAAAAGCGTTAAGAATTTCACGGTTCGCCGTGCTGACCGTTCAATCGTATGTGCGAGCGAGCGCCGAGCTAAGATACCTCTTATTGGTAACACAGTTCCTTTGGAACCGAGctgtctcttatacacctctccgagc                                                                                                                                                      >  1:116696/1‑277 (MQ=255)`  
                                                                                                                                                        `aaCGACTCACTAAAGGAGGTACACACCATGATGTACTTAATGCCATTACTCATCGTCATTGTAGGATGCCTTGCGCTCCACTGTAGCGATGATGATATGCCAGATGGTCACGCTTAATACGACTCACTAAAGGAGACA‑CTATATGTTTCGACTTCATTACAACAAAAGCGTTAAGAATTTCACGGTTCGCCGTGCTGACCGTTCAATCGTATGTGCGAGCGAGCGCCGAGCTAAGATACCTCTTATTGGTAACACAGTTCCTTTGGCACCGAGCGTCCACATCATTATCACCCGTGGTGAc                                                                                                                                                   <  1:272509/300‑1 (MQ=255)`  
                                                                                                                                                             `cTCACTAAAGGAGGTACACACCATGATGTACTTAATGCCATTACTCATCGTCATTGTAGGATGCCTTGCGCTCCACTGTAGCGATGATGATATGCCAGATGGTCACGCTTAATACGACTCACTAAAGGAGACA‑CTATATGTTTCGACTTCATTACAACAAAAGCGTTAAGAATTTCACGGTTCGCCGTGCTGACCGTTCAATCGTATGTGCGAGCGAGCGCCGAGCTAAGATACCTCTTATTGGTAACACAGTTCCTTTGGCACCGAGCGTCCACATCATTATCACCCctgtctcttatac                                                                                                                                              >  1:113587/1‑291 (MQ=255)`  
                                                                                                                                                               `cACTAAAGGAGGTACACACCATGATGTACTTAATGCCATTACTCATCGTCATTGTAGGATGCCTTGCGCTCCACTGTAGCGATGATGATATGCCAGATGGTCACGCTTAATACGACTCACTAAAGGAGACA‑CTATATGTTTCGACTTCATTACAACAAAAGCGTTAAGAATTTCACGGTTCGCCGTGCTGACCGTTCAATCGTATGTGCGAGCGAGCGCCGAGCTAAGATACCTCTTATTGGTAACACAGTTCCTTTGGCACCGAGCGTCcacctgtctcttatacacatctccgagcccc                                                                                                                                            >  1:4160/1‑273 (MQ=255)`  
                                                                                                                                                               `cACTAAAGGAGGTACACACCATGATGTACTTAATGCCATTACTCATCGTCATTGTAGGATGCCTTGCGCTCCACTGTAGCGATGATGATATGCCAGATGGTCACGCTTAATACGACTCACTAAAGGAGACA‑CTATATGTTTCGACTTCATTACAACAAAAGCGTTAAGAATTTCACGGTTCGCCGTGCTGACCGTTCAATCGTATGTGCGAGCGAGCGCCGAGCTAAGATACCTCTTATTGGTAACACAGTTCCTTTGGCACCGAGCGTCCAcatcctgtctcttatacacctctccgagc                                                                                                                                            >  1:209560/1‑276 (MQ=255)`  
                                                                                                                                                                 `cTAAAGGAGGTACACACCATGATGTACTTAATGCCATTACTCATCGTCATTGTAGGATGCCTTGCGCTCCACTGTAGCGATGATGATATGCCAGATGGTCACGCTTAATACGACTCACTAAAGGAGACA‑CTATATGTTTCGACTTCATTACAACAAAAGCGTTAAGAATTTCACGGTTCGCCGTGCTGACCGTTCAATCGTATGTGCGAGCGAGCGCCGAGCTAAGATACCTCTTATTGGTAACACAGTTCCTTTGGCACCGAGCGTCCACATCATTATCACCCGTGGTGACTTTGAGaaa                                                                                                                                          >  1:126652/1‑301 (MQ=255)`  
                                                                                                                                                                 `cTAAAGGAGGTACACACCATGATGTACTTAATGCCATTACTCATCGTCATTGTAGGATGCCTTGCGCTCCACTGTAGCGATGATGATATGCCAGATGGTCACGCTTAATACGACTCACTAAAGGAGACA‑CTATATGTTTCGACTTCATTACAACAAAAGCGTTAAGAATTTCACGGTTCGCCGTGCTGACCGTTCAATCGTATGTGCGAGCGAGCGCCGAGCTAAGATACCTCTTATTGGTAACACAGTTCCTTTGGCACCGAGCGTCCACATCATTATCACCCGTGGTGACTTTGAGaaa                                                                                                                                          >  1:199622/1‑301 (MQ=255)`  
                                                                                                                                                                       `gaggTACACACCATGATGTACTTAATGCCATTACTCATCGTCATTGTAGGATGCCTTGCGCTCCACTGTAGCGATGATGATATGCCAGATGGTCACGCTTAATACGACTCACTAAAGGAGACA‑CTATATGTTTCGACTTCATTACAACAAAAGCGTTAAGAATTTCACGGTTCGCCGTGCTGACCGTTCAATCGTATGTGCGAGCGAGCGCCGAGCTAAGATACCTATTATTGGTAACACAGTTCCTTTGGCACCGAGCGTCCACATCATTATCACCCGTGGTGACTTTgagctgtctctt                                                                                                                                    >  1:267405/1‑292 (MQ=255)`  
                                                                                                                                                                       `gaggTACACACCATGATGTACTTAATGCCATTACTCATCGTCATTGTAGGATGCCTTGCGCTCCACTGTAGCGATGATGATATGCCAGATGGTAACGCTTAATACGACTCACTAAAGGAGACA‑CTATATGTTTCGACTTCATTACAACAAAAGCGTTAAGAATTTCACGGTTCGCCGTGCTGACCGTTCAATCGTATGTGCGAGCGAGCGCCGAGCTAAGATACCTCTTATTGGTAACACAGTTCCTTTGGCACCGAGCGTCCACATCATTATCACCCGTGGTGACTTTGAGAAAGCAATa                                                                                                                                    >  1:332561/1‑301 (MQ=255)`  
                                                                                                                                                                          `gtaaaaacCATGATGTACTTAATGCCATTACTCATCGTCATTGTAGTATGCCTTGCGCTCCACTGTAGCGATGATGATATGCCAGATGGTCACGCTTAATACGACTCACTAAAGGAGACA‑CTATATGTTTCGACTTCATTACAACAAAAGCGTTAAGAATTTCACGGTTCGCCGTGCTGACCGTTCAATCGTATGTGCGAGCGAGCGCCGAGCTAAGATACCTCTTATTGGTAACACAGTTCCTTTGGCACCGAGCGTCCACATCATTATCACCCGTGGTGACTTTGAGAAAGCAATAGAc                                                                                                                                 <  1:142744/295‑1 (MQ=255)`  
                                                                                                                                                                          `gacagcacCATGATGTACTTAATGCCATTACTCATCGTCATTGTAGGATGCCTTGCGCTCCACTGTAGCGATGATGATATGCCAGATGGTCACGCTTAATACGACTCACTAAAGGAGACA‑CTATATGTTTCGACTTCATTACAACAAAAGCGTTAAGAATTTCACGGTTCGCCGTGCTGACCGTTCAATCGTATGTGCGAGCGAGCGCCGAGCTAAGATACCTCTTATTGGTAACACAGTTCCTTTGGCACCGAGCGTCCACATCATTATCACCCGTGGTGACTTTGAGAAAGCAATAGAc                                                                                                                                 <  1:276786/296‑1 (MQ=255)`  
                                                                                                                                                                           `tACACACCATGATGTACTTAATGCCATTACTCATCGTCATTGTAGGATGCCTTGCGCTCCACTGTAGCGATGATGATATGCCAGATGGTCACGCTTAATACGACTCACTAAAGGAGACA‑CTATATGTTTCGACTTCATTACAACAAAAGCGTTAAGAATTTCACGGTTCGCCGTGCTGACCGTTCAATCGTATGTGCGAGCGAGCGCCGAGCTAAGATACCTCTTATTGGTAACACAGTTCCTTTGGCACCGAGCGTCCACATCATTATCACCCGTGGTGACTTTGAGAAAGCAATAGACa                                                                                                                                <  1:103363/301‑1 (MQ=255)`  
                                                                                                                                                                               `cacCATGATGTACTTAATGCCATTACTCATCGTCATTGTAGGATGCCTTGCGCTCCACTGTAGCGATGATGATATGCCAGATGGTCACGCTTAATACGACTCACTAAAGGAGACA‑CTATATGTTTCGACTTCATTACAACAAAAGCGTTAAGAATTTCACGGTTCGCCGTGCTGACCGTTCAATCGTATGTGCGAGCGAGCGCCGAGCTAAGATACCTCTTATTGGTAACACAGTTCCTTTGGCACCGAGCGTCCACATCATTATCACCCGTGTTGACTTTGAGAAAGCAATAGACAAGaa                                                                                                                            >  1:194154/1‑301 (MQ=255)`  
                                                                                                                                                                                 `ccATGATGTACTTAATGCCATTACTCATCGTCATTGTAGGATGCCTTGCGCTCCACTGTAGCGATGATGATATGCCAGATGGTCACGCTTAATACTACTCACTAAAGGAGACA‑CTATATGTTTCGACTTCATTACAACACAAGCGTTAAGAATTTCACGGTTCGCCTTTCTGACCGTTCACTCGTATGTGCGTGCTAGCGCCGAGCTAAGATACCTTTTCTTGGTCACACAGTTCCTTTTGCACCTAGCGTCCACATCCTTCTCACTCTGTGTGACTTTGACCACGTAATAGCCAAGAAAc                                                                                                                          >  1:76964/1‑301 (MQ=255)`  
                                                                                                                                                                                 `ccATGATGTACTTAATGCCATTACTCATCGTCATTGTAGGATGCCTTGCGCTCCACTGTAGCGATGATGATATGCCAGATGGTCACGCTTAATACGACTCACTAAAGGAGACA‑CTATATGTTTCGACTTCATTACAACAAAAGCGTTAAGAATTTCACGGTTCGCCGTGCTGACCGTTCAATCGTATGTGCGAGCGAGCGCCGAGCTAAGATACCTCTTATTGGTAACACAGTTCCTTTGGCACCGAGCGTCCACATCATTATCACCCGTGGTGACTTTGAGAAAGCAATAGACAAGAAAc                                                                                                                          >  1:108010/1‑301 (MQ=255)`  
                                                                                                                                                                                   `atgatgTACTTAATGCCATTACTCATCGTCATTGTAGGATGCCTTGCGCTCCACTGTAGCGATGATGATATGCCAGATGGTCACGCTTAATACGACTCACTAAAGGAGACA‑CTATATGTTTCGACTTCATTACAACAAAAGCGTTAAGAATTTCACGGTTCGCCGTGCTGACCGTTCAATCGTATGTGCGAGCGAGCGCCGAGCTAAGATACCTCTTATTGGTAACACAGTTCCTTTGGCACCGAGCGTCCACATCATTATCACCCGTGGTGACTTTGAGAAAGCAATAGACAAGAAActg                                                                                                                        >  1:224903/1‑299 (MQ=255)`  
                                                                                                                                                                                     `gatgTACTTAATGCCATTACTCATCGTCATTGTAGGATGCCTTGCGCTCCACTGTAGCGATGATGATATGCCAGATGGTCACGCTTAATACGACTCACTAAAGGAGACA‑CTATATGTTTCGACTTCATTACAACAAAAGCGTTAAGAATTTCACGGTTCGCCGTGCTGACCGTTCAATCGTATGTGCGAGCGAGCGCCGAGCTAAGATACCTCTTATTGGTAACACAGTTCCTTTGGCACCGAGCGTCCACATCATTATCACCCGTGGTGACTTTGAGAAAGCAATAGACAAGAAACGTcc                                                                                                                      >  1:71967/1‑301 (MQ=255)`  
                                                                                                                                                                                          `aCTTAATGCCATTACTCATCGTCATTGTAGGATTCCTTGCGCTCCACTGTAGCGATGATGATATGCCAGATGATCACGCTTAATACGACTCACTAAAGGAGACA‑CTATATGTTTCGACTTCATTACAACAAAAGCGTTAAGAATTTCACGGTTCGCCGTGCTGACCGTTCAATCGTATGTGCGAGCGAGCGCCGAGCTAAGATACCTCTTATTGGTAACACAGTTCCTTTGGCACCGAGCGTCCACATCATTATCACCCGTGGTGACTTTGAGAAAGCAATAGACAAGAAACGTCCGGTTc                                                                                                                 >  1:84983/1‑301 (MQ=255)`  
                                                                                                                                                                                                    `aTTACTCATCGTCATTGTAGGATGCCTTGCGCTCCACTGTAGCGATGATGATATGCCAGATGGTCACGCTTAATACGACTCACTAAAGGAGACA‑CTATATGTTTCGACTTCATTACAACAAAAGCGTTAAGAATTTCACGGTTCGCCGTGCTGACCGTTCAATCGTATGTGCGAGCGAGCGCCGAGCTAAGATACCTCTTATTGGTAACACAGTTCCTTTGGCACCGAGCGTCCACATCATTATCACCCGTGGTGACTTTGAGAAAGCAATAGACAAGAAACGTCCGGTTCTTAGTGTGGc                                                                                                       <  1:312581/301‑1 (MQ=255)`  
                                                                                                                                                                                                      `tACTTATCTTAATTGTAGGATGCCTTGCGCTCTACGGTAGCGATGATGAGATGCCAGATGGTCACGCTTAATACGACTCACTAAAGGAGACA‑CTATATGTTTCGACTTCATTACAACAAAAGCGTTAAGAATTTCACGGTTCGCCGTGCTGACCGTTCAATCGTATGTGCGAGCGAGCGCCGAGCTAAGATACCTCTTATTGGTAACACAGTTCCTTTGGCACCGAGCGTCCACATCATTATCACCCGTGGTGACTTTGAGAAAGCAATAGACAAGAAACGTCCGGTTCTTAGTATGGCAg                                                                                                     <  1:151945/301‑1 (MQ=255)`  
                                                                                                                                                                                                      `tACTCATCGTCATTGTAGGATGCCTTGCGCTCCACTGTAGCGATGATGATATGCCAGATGGTCACGCTTAATACGACTCACTAAATGAGACA‑CTATATGTTTCGACTTCATTACAACAAAAGCGTTAAGAATTTCACGGTTCGCCGTGCTGACCGTTCAATCGTATGTGCGAGCGAGCGCCGAGCTAAGATACCTATTATTGGTAACGCAGTTCCTTTGGCACCGAGCGTCCACATCATTATCACCAGTGGTGACTTTGAGAAAGCAATAGACAAGAAACGTCCGGTTCTTAGTGTGGCAg                                                                                                     <  1:282640/301‑1 (MQ=255)`  
                                                                                                                                                                                                          `cATCTTCATTGTAGGATGCCTTGCGCTCCACTGTAGCGATGATGATATGCCAGATGGTCACGCTTATTCCGACTCACTAACGTCGGCA‑CTATATGTTTTGTCTTCATTACCACAAAGGCGTTAATAATTTCACGGTTCTCCGTGCTGACCGTTCAATCGTATGTGCGACCGAGCGCCGAGCTAAGATACCTCTTATTGGTAACACAGTTCCTTTGGCACCGAGCGTCGGCAGCATTATCACCCGTGGTGACTTTGAGAAAACAATAGACAAGCAACGTCCGGATCTTAGTGTGTCAGttcc                                                                                                 >  1:50832/1‑298 (MQ=255)`  
                                                                                                                                                                                                               `tCATTGTAGGATGCCTTGCGCTCCACTGTAGCGATGATGATATGCCAGATGGTCACGATTAATACGACTCACTAAAGGAGACA‑CTATATGTTTCGACTTCATTACAACAAAAGCGTTAAGAATTTCACGGTTCGCCGTGCTGACCGTTCAATCGTATGTGCGAGCGAGCGCCGAGCTAAGATACCTCTTATTGGTAACACAGTTCCTTTGGCACCGAGCGTCCACATCATTATCACCCGTGGTGACTTTGAGAAAGCAATAGACAAGAAACGTCCGGTTCTTAGTGTGGCAGTGACCCGCt                                                                                            >  1:5678/1‑301 (MQ=255)`  
                                                                                                                                                                                                                 `aagacaGGATGCCTTGCGCTCCACTGTAGCGATGATGATATGCCAGATGATCACGCTTAATACGACTCACTAAAGGAGACA‑CTATATGTTTCGACTTCATTACAACAAAAGCGTTAAGAATTTCACGGTTCGCCGTGCTGACCGTTCAATCGTATGTGCGAGCGAGCGCCGAGCTAAGATACCTCTTATTGGT‑ACACAGTTCCTTTGGCACCGAGCGTCCACATCATTATCACCCGTGGTGACTTTGAGAAAGCAATAGACAAGAAACGTCCGGTTCTTAGTGTGGCAGTGACCCGCTTcc                                                                                         <  1:160419/296‑1 (MQ=255)`  
                                                                                                                                                                                                                  `ttGTAGGATGCCTTGCGCTCCACTGTAGCGATGATGATATGCCAGATGGTCACGCTTAATACGACTCACTAAAGGAGACA‑CTATATGTTTCGACTTCATTACAACAAAAGCGTTAAGAATTTCACGGTTCGCCGTGCTGACCGTTCAATCGTATGTGCGAGCGAGCGCCGAGCTAAGATACCTCTTATTGGTAACACAGTTCCTTTGGCACCGAGCGTCCACATCATTATCACCCGTGGTGACTTTGAGAAAGCAATAGACAAGAAACGTCCGGTTCTTAGTGTGGCAGTGACCCGCTTcc                                                                                         <  1:232133/301‑1 (MQ=255)`  
                                                                                                                                                                                                                    `gTAGGATGCCTTGCGCTCCACTGTAGCGATGATGATATGCCAGATGGTCCCGCTTAATACGACTCACTAAAGGATACA‑CTATATGTTTCGAATTCATTACAACAAAAGCGTTAAACATTTCACGGTTAGACGTGCTAACAGTTAAGTCGTATGTGCGAGCGAGCGCCGCGCTAAGATACCTCTTATTGGTACCAAAGTTCCTTTGGCACCCAGTATCCACATCATTCTCACCCATAGTGCCTTTTAGCACGCATTAAACACGAAACGTCCGTTTCTTAGTGTGACAGTGACACGCTTCTCg                                                                                       >  1:325974/1‑301 (MQ=255)`  
                                                                                                                                                                                                                                `gcgcTCCACTGTAGCGATGATGATATGCCAGATGGTCACGCTTAATACGACTCACTAAAGGAGACA‑CTATATGTTTCGACTTCATTACAACAAAAGCGTTAAGAATTTCACGGTTCGCCGTGCTGACCGTTCAATCGTATGTGCGAGCGAGCGCCGAGCTAAGATACCTCTTATTGGTAACACAGTTCCTTTGGCACCGAGCGTCCACATCATTATCACCCGTGGTGACTTTGAGAAAGCAATAGACAAGAAACGTCCGGTTCTTAGTGTGGCAGTGACCCGCTTCCCGTTcgtcctgtct                                                                           >  1:146468/1‑296 (MQ=255)`  
                                                                                                                                                                                                                                 `cgcTCCACTGTAGCGATGATGATCTGCCAGATGGTCACGCTTAATACGACTCACTAAAGGAGACA‑CTATATGTTTCGACTTCATTACAACAAAAGCGTTAAGAATTTCACTGTTCGCCGTGCTGACCGTTCAATCGTATGTGCGAGCGAGCGCCGAGCTAAGATACCTCTTATTGGTAACACAGTTCCTTTGGCACCGAGCGTCCACATCATTATCACCCGTGGTGACTTTGAGAAAGCAATAGACAAGAAACGTCCGGTTCTTAGTGTGGCAGTGACCCGCTTCCCGTTCGTCCGTCTGt                                                                          <  1:234869/301‑1 (MQ=255)`  
                                                                                                                                                                                                                                   `cTCCACTGTAGCGATGATGATATGCCAGATGGTCACGCTTAATACGACTCACTAAAGGAGACA‑CTATATGTTTCGACTTCATTACAACAAAAGCGTTAAGAATTTCACGGTTCGCCGTGCTGACCGTTCAATCGTATGTGCGAGCGAGCGCCGAGCTAAGATACCTCTTATTGGTAACACAGTTCCTTTGGCACCGAGCGTCCACATCATTATCACCCGTGGTGACTTTGAGAAAGCAATAGACAAGAAACGTCCGGTTCTTAGTGTGGCAGTGACCCGCTTCCCGTTCGTCCGTCTGttt                                                                        >  1:279823/1‑300 (MQ=255)`  
                                                                                                                                                                                                                                     `ccACTGTAGCGATGATGATATGCCAGATGGTCACGCTTAATACGACTCACTAAAGGAGACA‑CTATATGTTTCGACTTCATTACAACAAAAGCGTTAAGAATTTCACGGTTCGCCGTGCTGACCGTTCAATCGTATGTGCGAGCGAGCGCCGAGCTAAGATACCTCTTATTGGTAACACAGTTCCTTTGGCACCGAGCGTCCACATCATTATCACCCGTGGTGACTTTGAGAAAGCAATAGACAAGAAACGTCCGGTTCTTAGTGTGGCAGTGACCAGCTTCCCGTTCGTCCGTCTGTTACt                                                                      >  1:295878/1‑301 (MQ=255)`  
                                                                                                                                                                                                                                        `cTGTAGCGATGATGATATGCCAGATGGTCACGCTTAATACGACTCACTAAAGGAGACA‑CTATATGTTTCGACTTCATTACAACAAAAGCGCTAAGAATTTCACGGTTCGCCGTGCTGACCGTTCAATCGTATGTGCGAGAGAGCGCCGAGCTAAGATACCTCTTATTGGTAACACAGTTCCTTTGGCACCGAGCGTCCACTTCATTATCACCAGTGGTGACTTTGAGAAAGCAATAGACAAGAAACGTCCGGATCTTAGTGTGGCAGTGACCCGCTACCCGTTCGTCAGTCTGTTACTCaa                                                                   >  1:334864/1‑301 (MQ=255)`  
                                                                                                                                                                                                                                                   `atgatATGCCAGATGGTAACGCTTAATACGACTCACTAAAGGAGACA‑CTATATGTTTCGACTTCATTCCAACAAAAGCGTTAAGAATTTCACGGTTCTCCGTGCTGACCGTTCAATCGTATGTGCGAGCGAGCGCCGAGCTAAGATACCTCTTATTGGTAACACAGTTCCTTTGGCACCGAGCGTCCACATCATTATCACCCGTGGTGACTTTGAGAAAGCAATAGACAAGAACCGGCCGGTTCTTAGTGTGGCAGTGACCCGCTTCCCGTTCGTCCGTCTGTTACTCAAACGAATCAagg                                                        <  1:206535/301‑1 (MQ=255)`  
                                                                                                                                                                                                                                                   `aagatATGCCAGATGGTCACGCTTAATACGACTCACTAACTGAGACA‑CTATTTGTTTCGACTTCATTACAACAAAAGCGTTAAGAATTTCACGGTTCGCCGTGCTGACCGTTCAATCGTATGTGCGAGCGAGCGCCGAGCTAAGATACCTCTTTTTTTTCACACAGTTCCTTTCGCACCGAGCGTCCACATCATTCTCACCCGTGGTGACGTTGAGAAAGCAATAGCCCATAAACGTCCGGTTCTTAGTTTGGCAGTGATCCGCTTCCCTTTCCTCCGTCTGTTACTCAAACGAATCAagg                                                        <  1:149115/299‑1 (MQ=255)`  
                                                                                                                                                                                                                                                    `tgatATGCCAGATGGTCACGCTTAATACGACTCACTAAAGGAGACA‑CTATATGTTTCGACTTCATTACAACAAAAGCGTTAAGAATTTCACGGTTCGCCGTGCTGACCGTTCAATCGTATGTGCGAGCGAGCGCCGAGCTAAGATACCTCTTATTGGTAACACAGTTCCTTTGGCACCGAGCGTCCACATCATTATCACCCGTGGTGACTTTGAGAAAGCAATAGACAAGAAACGTCCGGTTCTTAGTGTGGCAGTGACCCGCTTCCCGTTCGTCCGTATGTTACTCAAACGAATCAagga                                                       >  1:62083/1‑301 (MQ=255)`  
                                                                                                                                                                                                                                                     `gatATGCCAGATGGTCACGCTTAATACGACTCACTAAAGGAGACA‑CTATATGTTTCGACTTCATTACAACAAAAGCGTTAAGAATTTCACGGTTCTCCGTTCTGACCGTTCAATCGTATGTGCGAGCGAGCGCCGAGCTAAGCTACCTCTTATTGGTAACACAGTTCCTTTGGCACCGAGCGTCCACATCATTATCACCCGTGGTGACTTTGAGAAAGCAATAGACAAGAAACGTCCGGTTCTTAGTGTGGCAGTGACCCGCTTCCCGTTCGTCCGTCTGTTACTCCAACGAATCAaggag                                                      >  1:73967/1‑301 (MQ=255)`  
                                                                                                                                                                                                                                                     `gatATGCCAGATGGTCACGCTTAATACGACTCACTAAAGGAGACA‑CTATATGTTTCGACTTCATTACAACAAAAGCGTTAAGAATTTCACGGTTCGCCGTGCTGACCGTTCAATCGTATGTGCGAGCGAGCGCCGAGCTAAGATACCTCTTATTGGTAACACAGTTCCTTTGGCACCGAGCGTCCACATCATTATCACCCGTGGTGACTTTGAGAAAGCAATAGACAAGAAACGTCCGGTTCTTAGTGTGGCAGTGACCCGCTTCCCGTTCGTCCGTCTGTTACTCAAACGAATCAaggag                                                      >  1:240534/1‑301 (MQ=255)`  
                                                                                                                                                                                                                                                     `gatATGCCAGATGGTCACGCTTAATACGACTCACTAAAGGAGACA‑CTATATGTTTCGACTTCATTACAACAAAAGCGTTAAGAATTTCACGGTTCGCCGTGCTGACCGTTCAATCGTATGTGCGAGCGAGCGCCGAGCTAAGATACCTCTTATTGGTAACACAGTTCCTTTGGCACCGAGCGTCCACATCATTATCACCCGTGGTGACTTTGAGAAAGCAATAGACAAGAAACGTCCGGTTCTTAGTGTGGCAGTGACCAGCTTCCCGTTCGTCCGTCTGTTACTCAAACGAATCAaggag                                                      >  1:190885/1‑301 (MQ=255)`  
                                                                                                                                                                                                                                                       `tatGCCAGATGGTCACGCTTAATACGACTCACTAAAGGAGACA‑CTATATGTTTCGACTTCATTACAACAAAAGCGTTAAGAATTTCACGGTTCGCCGTGCTGACCGTTCAATCGTATGTGCGAGCGAGCGCCGAGCTAAGATACCTCTTATTGGTAACACAGTTCCTTTGGCACCGAGCGTCCACATCATTATCACCCGTGGTGACTTTGAGAAAGCAATAGACAAGAAACGTCCGGTTCTTAGTGTGGAAGTGACCCGCTTCCCGTTCGTCCGTCTGTTACTCAAACGAATCAaggaggc                                                    >  1:249384/1‑300 (MQ=255)`  
                                                                                                                                                                                                                                                         `tGCCAGATGGTCACGCTTAATACGACTCACTAAAGGAGACA‑CTATATGTTTCGACTTCATTACAACAAAAGCGTTAAGAATTTCACGGTTCGCCGTGCTGACCGTTCAATCGTATGTGCGAGCGAGCGCCGAGCTAAGATACCTCTTATTGGTAAAACAGTTCCTTTGGGACCGAGCGTCCACATCATTATCACCCGTGGTGACTTTGAGAACGCAATAGACAAGAAACGTCTGTTTCTTAGTGTGGCAGTGACCCGCTTCCCGTTCGTCCGTCTGTTACTCAAACGAATCAAGGAGgtgt                                                  >  1:299935/1‑301 (MQ=255)`  
                                                                                                                                                                                                                                                           `ccAGATGGTCACGCTTAATACGACTCACTAAAGGAGACA‑CTATATGTTTCGACTTCATTACAACAAAAGCGTTAAGAATTTCACGGTTCGCCGTGCTGACCGTTCAATCGTATGTGCGAGCGAGCGCCGAGCTAAGATACCTCTTATTGGTAACACAGTTCCTTTGGCACCGAGCGTCCACATCATTATCACCCGTGGTGACTTTGAGAAAGCAATAGACAAGAAACGTCCGGTTCTTAGTGTGGCAGTGACCCGCTTCCCGTTCGTCCGTCTGTTACTCAAACGAATCAAGGAGGTGTTc                                                <  1:275099/301‑1 (MQ=255)`  
                                                                                                                                                                                                                                                           `aagacaggTCACGCTTAATACGACTCACTAAAGGAGACC‑CTATATGTTTCGACTTCATTACAACAAAAGCGTTAAGAATTTCACGGTTCGCCGTGCTGACCGTTCAATCGTATGTGCGAGCGAGCGCCGAGCTAAGATACCTCTTATTGGTAACACAGTTCCTTTGGCACCGAGCGTCCACATCATTATCACCCGTGGTGACTTTGAGAAAGCAATAGACAAGAAACGTCCGGTTCTTAGTGTGGCAGTGACCCGCTTCCCGTTCGTCCGTCTGTTACTCAAACGAATCAAGGAGGTGTTc                                                <  1:106099/295‑1 (MQ=255)`  
                                                                                                                                                                                                                                                                `tGGTCACGCTTAATACGACTCACTAAAGGAGACA‑CTATATGTTTCGACTTCATTACAACAAAAGCGTTAAGAATTTCACGGTTCGCCGTGCTGACCGTTCAATCGTATGTGCGAGCGAGCGCCGAGCTAAGATACCTCTTATTGGTAACACAGTTCCTTTGGCACCGAGCGTCCACATCATTATCACCCGTGGTGACTTTGAGAAAGCAATAGACAAGAAACGTCCGGTTCTTAGTGTGGCAGTGACCCGCTTCCCGTTCGTCCGTCTGTTACTCAAACGAATCAAGGAGGTGTTCTGATg                                           <  1:166659/301‑1 (MQ=255)`  
                                                                                                                                                                                                                                                                `agttaactggtAATACGACTCACTAAAGGAGACA‑CTATATGTTTCGACTTCATTACAACAAAAGCGTTAAGAATTTCACGGTTCGCCGTGCTGACCGTTCAATCGTATGTGCGAGCGAGCGCCGAGCTAAGTTACCTATTATTGGTAACACAGTTCCTTTGGCACCGCGCGTCCACATCATTATTACCCGTGTTGACTTTGAGAATGCAATATACAAGAATCGTCCGGTTCTTAGTGTGGCAGTGACACGCTTCCCGTTCGTCCGTCTGTTACTCAAATGAATCCAGGAGGTGTCTTGATg                                           >  1:238446/11‑301 (MQ=255)`  
                                                                                                                                                                                                                                                                `aggtaaacactAATACGACATACGAAGGGAGACA‑CTATATGTGACAACAGCATTACAACAAAAGCGTTAAGAATTTCACGGTTCTGCGTGCGTACCGTTCAATCGTATGTGCGATCGAGCGCCGAGCTAAGAAACCTCTTATTGGTAACACAGTGCCTTTGGCACCGAGCGTCAACATCATTATCACCCGTGGTGACTTTGAGAAAGCAATAGACAAGAAACGTCCGGTTCTTAGTGTGGCAGTGACCCGCTTCCCGTTCGTCCGTATGTTACTCAAACGAATCAAGGAGGTGTTCTGATg                                           <  1:44757/291‑1 (MQ=255)`  
                                                                                                                                                                                                                                                                     `aaagagacagcGACTCACTAAAGGAGACA‑CTATATGTTTCGACTTCATTACAACAAAAGCGTTAAGAATTTCACGGTTCGCCGTGCTGACCGTTCAATCGTATGTGCGAGCGAGCGCCGAGCTAAGATACCTCTTATTGGTAACACAGTTCCTTTGGCACCGAGCGTCCACATCATTATCACCCGTGGTGACTTTGAGAAAGCAATAGACAAGAAACGTCCGGTTCTTAGTGTGGCAGTGACCCGCTTCCCGTTCGTCCGTCTGTTACTCAAACGAATCAAGGAGGTGTTCTGATGGGACt                                      <  1:185456/291‑1 (MQ=255)`  
                                                                                                                                                                                                                                                                      `cGCTTAATACGACTCACTAAAGGAGACA‑CTATATGTTTCGACTTCATTACAACAAAAGCGTTAAGAATTTCACGGTTCGCCGTGCTGACCGTTCAATTGTATGTGCGAGCGAGCGCCGAGCTAAGATACCTCTTATTGGTAACACAATTCCTTTGGCACCGAGCGTCCACATCATTATCACCCGTGGTGACTTTGAGAAAGCAATAGACAAGAAACGTCCGGTTCTTATTGTGGCAGTGACCCGCTTCCCGTTCTTCCGTCTGTTACTCAAACGAATCAAGGAGGTTTTCTGATGGGATTg                                     >  1:264836/1‑301 (MQ=255)`  
                                                                                                                                                                                                                                                                      `cGCTTAATACGACTCACTAAAGGAGACA‑CTATATGTTTCGACTTCATTACAACAAAAGCGTTAAGAATTTCACGGTTCGCCGTGCTGACCGTTCAATCGTATGTGCGAGCTAGCGCCGAGCTAAGATACCTCTTATTGGTAACACAGTTCCTTTGGCACCGAGCGTCCACATCATTATCACCCGTGGTGACTTTGAGAAAGCAATAGACAAGAAACGTCCGGTTCTTAGTGTGGCAGTGACCCGCTTCCCGTTCGTCCGTCTGTTACTCAAACGAATCAAGGAGGTGTTCTGATGGGACTg                                     <  1:12035/301‑1 (MQ=255)`  
                                                                                                                                                                                                                                                                      `cGCTTAATACGACTCACTAAAGGAGACA‑CTAGATGTTTCGACTTCATTACAACAAAAGCGTTAAGAATTTCACGGTTCGCCGTGCTGACCGTTCAATCGTATGTGCGAGCGAGCGCCGAGCTAAGATACCTCTTATTGGTAACACAGTTCCTTTGGCACCGAGCGTCCACATCATTATCACCCGTGGTGACTTTGAGAAAGCAATAGACAAGAAACGTCCGGTTCTTAGTGTGGCAGTGACCCGCTTCCCGTTCGTCCGTCTGTTACTCAAACGAATCAAGGAGGTGTTCTGATGGGACTg                                     <  1:292496/301‑1 (MQ=255)`  
                                                                                                                                                                                                                                                                         `ttAATACGACTCACTAAAGGAGACA‑CTATATGTTTCGACTTCATTACAACAAAAGCGTTAAGAATTTCACGGTTCGCCGTGCTGACCGTTCAATCGTATGTGCGAGCGAGCGCCGAGCTAAGATACCTCTTATTGGTAACACAGTTCCTTTGGCACCGAGCGTCCACATCATTATCACCCGTGGTGACTTTGAGAAAGCAATAGACAAGAAACGTCCGGTTCTTAGTGTGGCAGTGACCCGCTTCCCGTTCGTCCGTCTGTTACTCAAACTAATCAAGGAGGTGTTCTGATGGGACTGTTa                                  <  1:35359/301‑1 (MQ=255)`  
                                                                                                                                                                                                                                                                              `aCGACTCACTAAAGGAGACA‑CTATATGTTTCGACTTCATTACAACAAAAGCGTTAAGAATTTCACGGTTCGCCGTGCTGACCGTTCAATCGTATGTGCGAGCGAGCGCCGAGCTAAGATACCTCTTATTGGTAACACAGTTCCTTTGGCACCGAGCGTCCACATCATTATCACCCGTGGTGACTTTGAGAAAGCAATAGACAAGAAACGTCCGGTTCTTAGTGTGGCAGTGACCCGCTTCCCGTTCGTCCGTCTGTTACTCAAACGAATCAAGGAGGTGTTCTGATGGGACTGTTAGATgg                             <  1:319160/301‑1 (MQ=255)`  
                                                                                                                                                                                                                                                                              `aCGACTCACTAAAGGAGAAA‑ATAGATGTTGCGACGTCAGTACAACAAAAGCGTTAAGAATTTCACGGTTCGCCGTGCTGACCGTTCAATCGTATGTGCGAGCGAGCGCCGAGCTAAGATACCTCTTATTGGTAAAACAGTTCCTTTGGCACCGAGCGTCCACATCATTATCACCCGTGGTGACTTTGAGAAAGCAATAGACAAGAAACGTCCGGTTCTGAGTGTGGCAGTGAACCGCTTCCCGTTCGTCCGTCTGTTACTCAAACGAATCAAGGAGGTGTTCTGATGGGACTATTAGATgg                             <  1:155602/301‑1 (MQ=255)`  
                                                                                                                                                                                                                                                                              `aCGACTAACTAAAGGAGACA‑CTATATTTTTCGACTTCATTACAACAAAAGCGTTAAGAATTTCACGGTTCGCCGTGCTGACCGTTCAATCGTATGTGCGAGCGAGCGCCGAGCTAAGATACCTCTTATTGGTAACACAGTTCCTTTGGCACCGAGCGTCCACATCATTATCCCCCGTGGTGACTTTGAGAAAGCAATAGACAAGAAACGTCCGGTTCTTAGTGTGGCAGTGACCCGCTTCCCGTTCGTCCGTCTGTTACTCAAACGAATCAAGGAGGTGTTCTGATGGGACTGTTAGATgg                             <  1:38381/301‑1 (MQ=255)`  
                                                                                                                                                                                                                                                                               `gagatgtgtataagagaCAGCTATATGTTTCGACTTCATTACAACAAAAGCGTTAAGAATTTCACGGTTCGCCGTGCTGACCGTTCAATCGTATGTGCGAGCGAGCGCCGAGCTAAGATACCTCTTATTGGTAACACAGTTCCTTTGGCACCGAGCGTCCACATCATTATCACCCGTGGTGACTTTGAGAAAGCAATAGACAAGAAACGTCCGGTTCTTAGTGTGGCAGTGACCCGCTTCCCGTTCGTCCGTCTGTTACTCAAACGAATCAAGGAGGTGTTCTGATGGGACTGTTAGATgg                             <  1:171683/288‑1 (MQ=255)`  
                                                                                                                                                                                                                                                                                    `cACTAAAGGAGACA‑CTATATGTTTCGACTTCATTACAACAAAAGCGTTAAGAATTTCACGGTTCGCCGTGCTTACCGTTCAATCGTATGTGCGAGCGAGCGCCGAGCTAAGATACCTCTTATTGGTAACACAGTTCCTTTGGCACCGAGCGTCCACATCATTATCACCCGTGGTGACTTTGAGAAAGCAATATACAAGAAACGTCCGGTTCTTAGTGTGGCAGTGACCCGCTTCCCGTTCGTCCGTCTGTTACTCAAACGAATCAAGGAGGTGTTCTGactgtctcttatacacatctccg                       >  1:247395/1‑279 (MQ=255)`  
                                                                                                                                                                                                                                                                                     `aaaaaaGGAGAAA‑ATATAAGTATCGAATGAATTAAAACAAAAGCGTTAAGAATTTAACGGGTCGCCGTGAGGACAGTTCAATAGTATGTGCGAGCGAGGGCTGAGCTAAGAGAACTCGTATTGGTAACACAGTTCCTTTGGCACCGAGAGTCCACAGCATTATCACCCGTGGTGACTTTGAGAAAGGAATAGACAAGAAAAGTCCGGTTGTGAGTGTGGAAGTGACAAGCTTCCCGTTCGTCCGTCTGTTACTCAAACGAATCAAGGAGGTGTTCTGATGGGACTGTTAGATGGTGAAGcc                      <  1:81896/298‑1 (MQ=255)`  
                                                                                                                                                                                                                                                                                        `aaaGGAGACA‑CTATATGTTTCGACTTCATTACAACAAAAGCGTTAAGAATTTCACGGTTCGCCGTGCTGACCGTTCAATCGTATGTGCGAGCGAGCGCCGAGCTAAGATACCTCTTATTGGTAACACAGTTCCTTTGGCACCGAGCGTCCACATCATTATCACCCGTGGTGACTTTGAGAAAGCAATAGACAAGAAACGTCCGGTTCTTAGTGTGGCAGTGACCCGCTTCCCGTTCGTCCGTCTGTTACTCAAACGAATCAAGGAGGTGTTCTGATgctgtctcttatacacatctccgag                   >  1:307340/1‑277 (MQ=255)`  
                                                                                                                                                                                                                                                                                        `aaaGGAGACA‑CTATATGTTTCGACTTCATTACAACAAAAGCGTTAAGAATTTCACGGTTCGCCGTGCTGACCGTTCAATCCTATGTGCTAGCGAGCGCCGAGCTAAGATACCTCTTATTGGTAACACAGTTCCTTTGGCACCGAGCGTCCACATCATTATCACCCGTGGTGACTTTGAGCAAGCAATAGACAAGAAACGTCCGGTTCTTAGTGTGGCAGTGACCCGCTTCCCGTTCGTCCGTCTGTTACTCAAACGAATCAAGGCGGTGTTCTGCTGGGACTGTTAGactgtctcttatac                   >  1:177922/1‑288 (MQ=255)`  
                                                                                                                                                                                                                                                                                         `aaGGAGACA‑CTATATGTTTCGACTTCATTACAACAAAAGCGTTAAGAATTTCACGGTTCGCCGTGCTGACCGTTCAATCGTATGTGCGAGCGAGCGCCGAGCTAAGATACCTCTTATTGGTAACACAGTTCCTTTGGCACCGAGCGTCCACATCATTATCACCCGTGGTGACTTTGAGAAAGCAATAGACAAGAAACGTCCGGTTCTTAGTGTGGCAGTGACCCGCTTCCCGTTCGTCCGTCTGTTACTCAAACGAATCAAGGAGGGGTGCTGATGGGCATGGTAGATGGTGAAGCCTggg                  >  1:286939/1‑301 (MQ=255)`  
                                                                                                                                                                                                                                                                                          `aGGCGACA‑CTATATGTTTCGACTTCATTACAACAAAAGCGTTAAGAATTTCACGGTTCGCCGTGCTGACCGTTCAATCGTATGTGCGAGCGAGCGCCGAGCTAAGATACCTCTTATTGGTAACACAGTTCCTTTGGCACCGAGCGTCCACATCATTATCACCCGTGGTGACTTTGAGAAAGCAATAGACAAGAAACGTCCGGTTCTTAGTGTGGCAGTGACCCGCTTCCCGTTCGTCCGTCTGTTACTCAAACGAATCAAGGAGGTGTTCTGATGGGACTGTTAGATGGTGAAGCCTGGGa                 <  1:229627/301‑1 (MQ=255)`  
                                                                                                                                                                                                                                                                                          `aGGAGACA‑CTATATGTTTCGACTTCATTACAACAAAAGCGTTAAGAATTTCACGGTTCGCCGTGCTGACCGTTCAATCGTATGTGCGAGCGAGCGCCGAGCTAAGATACCTCTTATTGGTAACACAGTTCCTTTGGCACCGAGCGTCCACATCATTATCACCCGTGGTGACTTTGAGAAAGCAATAGACAAGAAACGTCCGGTTCTTAGTGTGGCAGTGACCCGCTTCCCGTTCGTCCGTCTGTTACTCAAACGAATCAAGGAGGTGTTCTGATGGGACTGTTAGAtctgtctcttataca                 >  1:216135/1‑287 (MQ=255)`  
                                                                                                                                                                                                                                                                                                `ca‑cTATATGTTTCGACTTCATTACAACAAAAGCGTTAAGAATTTCACGGTTCGCCGTGCTGACCGTTCAATCGTATGTGCGAGCGAGCGCCGAGCTAAGATACCTCTTATTGGTAACACAGTTCCTTTGGCACCGAGCGTCCACATCATTATCACCCGTGGTGACTTTGAGAAAGCAATAGACAAGAAACGTCCGGTTCTTAGTGTGGCCGTGACCCGCTTCCCGTTCGTCCGTCTGTTACTCAAACGAATCAAGGAGGTGTTCTGATTGGACTGTTCGATGGTGAAGCCTGGGAAAAAGa           >  1:187816/1‑301 (MQ=255)`  
                                                                                                                                                                                                                                                                                                 `a‑cTATATGTTTCGACTTCATTACAACAAAAGCGTTAAGAATTTCACGGTTCGCCGTGCTGACCGTTCAATCGTATGTGCGAGCGAGCGCCGAGCTAAGATACCTCTTATTGGTAACACAGTTCCTTTGGCACCGAGCGTCCACATCATTATCACCCGTGGTGACTTTGAGAAAGCAATAGACAAGAAACGTCCGGTTCTTAGTGTGGCAGTGACCCGCTTCCCGTTCGTCCGTCTGTTACTCAAACGAATCAAGGAGGTGTTCTGATGGGACTGTTAGATGGTGAAGCCTGGGAAAAAGaa          >  1:196425/1‑301 (MQ=255)`  
                                                                                                                                                                                                                                                                                                      `tatTTTTCGACTTCATTACAACAAAAGCGTTAAGAATTTCACGGTTCGCCGTGCTGACCGTTCAATCGTATGTGCGAGCGAGCGCCGAGCTAAGATACCTCTTATTGGTAACACAGTTCCTTTGGCACCGAGCGTCCACATCATTATCACCCGTGGTGACTTTGAGAAAGCAATAGACAAGAAACGTCCGGTTCTTAGTGTGGCAGTGACCCGCTTCCCGTTCGTCCGTCTGTTACTCAAACGAATCAATGAGGTGTTCTGATGGGACTGTTAGATGGTGAAGCCTGGGAAAAAGAAAAcc      <  1:303712/301‑1 (MQ=255)`  

                                                                                                                                                                                                                                                                                                             `|`                                                                                                                                                                                                                                                                                                           
`TTAAGAAGGTTGGTAAATTCCTTGCGGCTTTGGCAGCTATCCTGACGCTTGCGTATATTCTTGCGGTATACCCTCAAGTAGCACTAGTAGTAGTTGGCGCTTGTTACTTAGCGGCAGTGTGTGCTTGCGTGTGGAGTATAGTTAACTGGTAATACGACTCACTAAAGGAGGTACACACCATGATGTACTTAATGCCATTACTCATCGTCATTGTAGGATGCCTTGCGCTCCACTGTAGCGATGATGATATGCCAGATGGTCACGCTTAATACGACTCACTAAAGGAGACA‑CTATATGTTTCGACTTCATTACAACAAAAGCGTTAAGAATTTCACGGTTCGCCGTGCTGACCGTTCAATCGTATGTGCGAGCGAGCGCCGAGCTAAGATACCTCTTATTGGTAACACAGTTCCTTTGGCACCGAGCGTCCACATCATTATCACCCGTGGTGACTTTGAGAAAGCAATAGACAAGAAACGTCCGGTTCTTAGTGTGGCAGTGACCCGCTTCCCGTTCGTCCGTCTGTTACTCAAACGAATCAAGGAGGTGTTCTGATGGGACTGTTAGATGGTGAAGCCTGGGAAAAAGAAAACCCGCC  >  NC_001604.1/7612‑8209`

  

Alignment Legend

Aligned base mismatch/match (shaded by quality score): `ATCG/ATCG` `< 3 ≤` `ATCG/ATCG` `< 11 ≤` `ATCG/ATCG` `< 23 ≤` `ATCG/ATCG` `< 35 ≤` `ATCG/ATCG` `< 38 ≤` `ATCG/ATCG`

Unaligned base: `atcg`    Masked matching base: `atcg`    Alignment gap: `‑`    Deleted base: `‑`

Reads not counted as support for junction

read\_name Not counted due to insufficient overlap past the breakpoint.

read\_name Not counted due to not crossing MOB target site duplication.
